# Supplementary material for: Herring Milt Protein Hydrolysate Improves Insulin Resistance in High-Fat-Diet-Induced Obese Male C57BL/6J Mice
Source: Mar Drugs. 2019 Aug 3;17(8):456. doi: 10.3390/md17080456 (PMC6724050; doi:10.3390/md17080456)
Supplement: Supplementary file 1 [file marinedrugs-17-00456-s001.pdf]

Supplementary materials

Table S1. Diet composition and calorie information

Ingredient[#]	LFC*	HFC	HPH15	HPH35	HPH70
Diet composition					
Casein (80 Mesh)	200	200	170	130	60
L-Cystine	3	3	3	3	3
Corn Starch	315	0	0	0	0
Maltodextrin 10	35	125	125	125	125
Sucrose	350	68.8	68.8	68.8	68.8
Herring Milt Hydrolysate	0	0.00	42.4	98.9	197.7
Cellulose, BW200	50	50	50	50	50
Lard (96%)	20	245	240.4	234.3	223.6
Soybean Oil	25	25	25	25	25
Mineral Mix S10026	10	10	10	10	10
DiCalcium Phosphate	13	13	13	13	13
Calcium Carbonate	5.5	5.5	5.5	5.5	5.5
Potassium Citrate, 1 H2O	16.5	16.5	16.5	16.5	16.5
Vitamin Mix V10001	10	10	10	10	10
Choline Bitartrate	2	2	2	2	2
FD&C Blue Dye #1	0.05	0.05	0.05	0.05	0.05
Total	1055	774	782	792	810
Calorie information					
Protein (% kcal)	20	20	20	20	20
Fat (% kcal)	10	60	60	60	60
Carbohydrate (% kcal)	70	20	20	20	20
Energy density (kcal/g)	3.82	5.21	5.21	5.21	5.21

*LFC, low-fat diet; HFC, high-fat diet; HPH15, HPH35 and HPH70, HFC with 15%, 35% and 70% casein replaced, respectively, by the same amount of protein from herring milt protein hydrolysate.

[#]Diet formulae of LFC and HFC and the ingredients were obtained from the Research Diets, New Brunswick, NJ, USA.
